# The Role of *PON1* Variants in Disease Susceptibility in a Turkish Population

**DOI:** 10.1055/s-0040-1715568

**Published:** 2020-08-31

**Authors:** Mahmoud Abudayyak, Tuğçe Boran, Rumeysa Tukel, Ezgi Oztas, Gül Özhan

**Affiliations:** 1 Department of Pharmaceutical Toxicology, Faculty of Pharmacy, Istanbul University, Istanbul, Turkey

**Keywords:** paraoxonase-1, hypothyroidism, pancreatitis

## Abstract

Paraoxonase 1 (PON1) enzyme plays a major role in antioxidant defense and protects the cells against reactive species. The most common *PON1* Q192R and L55M polymorphisms are responsible for a wide variation of PON1 activity, which showed an up to 13-fold interindividual variation among the same genotype. *PON1* genotypes were evaluated with the development of pancreatitis, colorectal cancer, and hypothyroidism in a hospital-based, case-control study. Individuals with rs662 *G* allele had a two-fold risk of developing hypothyroidism. A weak association was found between rs854560 *T* allele and pancreatitis. The results were preliminary. Further studies with a larger number and detailed biochemical parameters are needed.

## Introduction

Paraoxonase (PON) is one of the more important antioxidants and antiatherogenic enzymes. It is mainly synthesized in the liver and metabolizes organophosphate, carbamate, aromatic carboxylic acid ester, unsaturated aliphatic ester, cyclic carbonate, and lactone compounds. It plays a significant role in the detoxification of nerve gases such as sarin, soman, and tabun. 1 2 3 The *PON* family consists of three genes: *PON1, PON2, PON3* , with each encoding a unique protein. The *PON* genes, located adjacent to each other on chromosome 7 in humans, show approximately 65% similarity in amino acids. 4 5 *PON1* stimulates high-density lipoprotein (HDL)-mediated cholesterol flow, protects against oxidative damage, and has peroxidase-like activity. Proteins encoded by the *PON1* and *PON3* are found in the blood circulation, 6 while the intracellular enzyme encoded by *PON2* is not found in the circulation. However, all the enzymes prevent oxidation of lipids 7 8 and are important for components of the antioxidant system in humans. Therefore, dysfunction in these enzymes can be associated with some diseases related to oxidative stress such as cancer and cardiovascular disease. 9

**Fig. 1:**
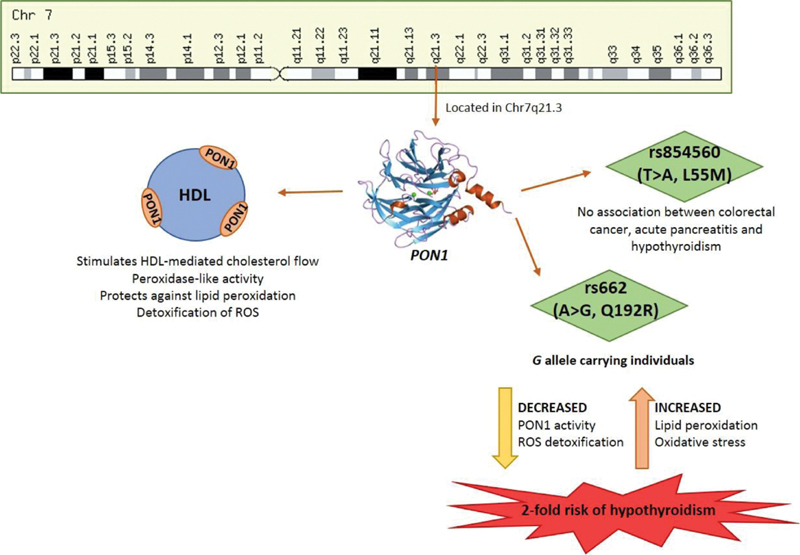
Relation between the rs854560 and rs662 polymorphisms of *PON1* and disease development.

*PON1* variation is the most studied member of the *PON* gene family. The coding region of *PON1* has two polymorphic sites, which are leucine (L)/methionine (M) at position 55 (L55M, rs854560) and glutamine (Q)/arginine (R) at position 192 (Q192R, rs662). 10 The rs854560 polymorphism leads to changes in the serum enzyme concentration. PON1 enzyme concentration is higher in AA genotypes than TT genotypes. 11 12 13 The rs662 polymorphism affects substrate specificity of the PON1 hydrolytic activity. Diazinon, soman, and sarin are hydrolyzed more efficiently in 192Q isoform of the PON1 enzyme, which is encoded *A* allele, whereas paraxon is hydrolyzed at a higher rate than 192R isoform of PON1 enzyme, which is encoded by *G* allele. Also, *A* allele prevents the accumulation of lipid peroxides more than *G* allele 14 15 ( Fig. 1 ).

PON1 enzymatic activity is also affected by many physiological and pathological factors such as pregnancy, aging, diabetes, and renal disease. 16 PON1 activity has been found decreased in type 1 and 2 diabetes as well as metabolic syndrome, which could be related with reduced secretion of insulin and decreased ability of HDL. 17 Some researchers reported that *PON1* polymorphisms could be related to diseases associated with oxidative stress-related inflammation like acute pancreatitis and neurodegenerative diseases such as Alzheimer's. 18 19 Oxidative stress lead to reduction in PON1 and PON3 enzymatic activity, while PON2 enzyme activity increases under oxidative stress conditions. 10

Oxidative stress plays a key role in the pathogenesis of several diseases via numerous pathways. Although thyroid hormones mainly regulate protein, vitamin and antioxidant enzymes synthesis and degradation, they also play a role in reactive oxygen species (ROS) production. 20 ROS are highly reactive molecules that mainly attack membrane lipids, and inadequate removal of ROS could result in lipid peroxidation, metabolic dysfunctions, damaged macromolecules and eventually cell death.

Since *PON1,* a known antioxidant, has a peroxidase-like activity, it could prevent oxidative stress in hypothyroidism. However, several reports indicated elevated oxidative stress parameters 21 22 and decreased PON1 activity 23 in hypothyroid patients. It is well-known that increased level of ROS is related to many cancers including colorectal cancers. PON1 enzyme is a phase 1 detoxification enzyme with a wide range of substrate specificity. Therefore, PON1 enzyme activities may alter ROS production or the rate of chemical metabolism, and this may influence the risk of colorectal cancer. 24 Acute pancreatitis is an inflammatory disease mediated by cytokines, bioactive lipids, and oxidative stress. 25 Paraoxonase activity is regulated by interleukin-1 and tumor necrosis factor-a 26 ; besides oxidative response, *PON1* may also be a part of the inflammatory response.

Based on the significant role of *PON1* in oxidant-antioxidant balance, the present study aimed to evaluate the relationship between *PON1* polymorphism and disease susceptibility in a small-sized patient population with hypothyroidism, colorectal cancer, and pancreatitis.

## Material and Methods

### Sample Collection

The study was conducted as a cross-sectional study with Turkish participants recruited from Istanbul University Medical Faculty between February 2017 and March 2018. The effects of the *PON1* polymorphisms on hypothyroidism, colorectal cancer, and pancreatitis susceptibility were evaluated among a total of 200 participants who volunteered. Fifty patients with secondary hypothyroidism caused by total thyroidectomies; 50 patients with histopathologically confirmed colorectal cancer; and 50 patients clinically diagnosed with mild or severe acute pancreatitis were included. Fifty ethnic, age and gender-matched controls who had no history of these conditions were selected randomly from hospital patients. The sample size was proper to evaluate the relationship between selected single nucleotide polymorphisms (SNPs) and diseases based on power analysis ( http://osse.bii.a-star.edu.sg ). The study was approved by the ethical committee of Istanbul University (2017/1370) and performed in accordance with the Helsinki Declaration of 1975. All participants were provided informed consent. Demographic and anthropometric factors (ethnicity, age, body mass index [BMI, kg/cm ^2^ ]) were assessed using a short questionnaire.

### Genotyping

Genomic DNA was extracted from venous blood samples by High Pure PCR Template Preparation Kit (Roche, Germany) and kept at 4°C. Genotyping was performed on real-time PCR platform (LightCycler 480, Roche, Germany) using LightCycler FastStart DNA Master HybProbe and Roche LightSNP assay probes (Roche, Germany) according to the manufacturer's instructions. In a final volume of 20 µL reaction mix per sample, the following mixtures was added: 1X FastStart DNA Master Mix, 2 mM MgCl _2_ , 0.2 µM LightSNP HybProbe, appropriate amount of PCR grade water, and 500 ng DNA sample. In each run, sterile water and a known genotyped sample were used as negative and positive controls, respectively, to achieve 100% concordance. Details of custom-designed LightSNiP assay probes were summarized in Table 1 .

**Table 1:** Reference sequences of custom-designed LightSNiP assay probes

LightSNiP	Reference sequence	Melting temperatures
rs662 **(575A** **>** **G)**	TCACTATTTTCTTgACCCCTACTTAC [A/g] ATCCTgggAgATgTATTTgggTTTA	52.56 °C for allele [G] 60.28 °C for allele [A]
rs854560 **(163T** **>** **A)**	AgCCAgTCCATTAggCAgTATCTCCA[A/T] gTCTTCAgAgCCAgTTTCTgCCAgA	60.23 °C for allele [A] 65.50 °C for allele [T]

Abbreviation: rs: reference SNP number; alleles in the square brackets indicates the polymorphisms.

### Statistical Analysis

All statistical analyses were performed using Statistical Package for Social Sciences (SPSS) software (Version 20, Chicago, USA). The Hardy–Weinberg Equilibrium (HWE) analysis was performed to compare the observed and expected genotype frequencies of subject by using the Chi-squared (χ ^2^ ) test. Data comparisons were done by using Fisher's exact test. The odds ratios (OR) and 95% confidence intervals (CI) were estimated to evaluate the association between cases and controls. A two-tailed value of *p* < 0.05 was considered a statistically significant difference.

## Results

In the present study, two SNPs of the *PON1* , rs662 and rs854560, were genotyped in a Turkish population. The cases and controls were evaluated to determine the effects of *PON1* polymorphisms on hypothyroidism, colorectal cancer, and pancreatitis susceptibility. All participants were Caucasian, and the characteristics of the studied population were summarized in Table 2 . Gender distribution was almost the same in all groups except hypothyroidism where females had a representation of 91%. In disease groups, patients who were ≥ 50 years old represented at least 61.3% of cases, whereas the control group was relatively young with volunteers who are ≥ 50 years old accounting for 44.5%. Nonsmokers represented at least 60% of all patients; however, approximately one third of the patients were overweight. As shown in Table 3 , the genotype distribution was found to be consistent with the HWE among all participants ( *p* > 0.05), indicating that the studied population was randomized and unbiased. The minor allele frequencies of the studied SNPs were found notably similar to Europeans 15 17 27 ; however, highly different from Asians. 28 29

**Table 2:** Demographic and anthropometric factors of cases and controls

Variable	Percentile (%)			
	Control ( *n* = 50)	Hypothyroidism ( *n* = 50)	Colorectal cancer ( *n* = 50)	Pancreatitis ( *n* = 50)
Gender	
Male	44.4	8.2	59.4	51.3
Female	55.6	91.8	40.6	48.7
Age	
≥ 50	44.5	78.6	61.3	72.9
< 50	55.5	21.4	38.7	27.1
BMI	
□ 25	18.6	31.8	30	48.6
> 25	81.4	68.2	70	51.4
Smoking status	
Nonsmoking	62.9	86.4	90	59.4
Smoking	37.1	13.6	10	40.6

Abbreviation: BMI, body mass index (kg/m ^2^ ).

**Table 3:** Genotype distribution of rs662 and rs854560 in the Turkish population

SNPs	Genotypes	Observed (n)	Expected (n)	* χ ^2^*	*p* -Value
rs662 ( *575A* *>* *G* )	A/A	109	103	4.13	0.134
	A/G	69	54		
	G/G	22	16		
rs854560 ( *163T* *>* *A* )	A/A	83	79	4.09	0.167
	T/A	85	93		
	T/T	32	28		

Abbreviations: n, number of subjects; rs, reference SNP number; SNPs, single nucleotide polymorphisms; χ ^2^ , Chi-square.

Note: *p* <0.05, significance level.

The dominant model was used to evaluate whether *PON1* polymorphisms increase the risk of hypothyroidism, colorectal cancer, or pancreatitis. The genotype distributions, allele frequencies and estimated ORs with 95% CI among all cases and controls are summarized in Table 4 . The genotyping analysis based on a dominant model showed that individuals with *G* allele of rs662 had an approximately two-fold risk of developing hypothyroidism (OR = 2.05; 95% CI = 1.05 to 3.97; *p* = 0.047). In addition, it was observed that individuals with *T* allele of rs854560 had an approximately 1.5-fold risk of developing pancreatitis (OR = 1.44; 95% CI = 0.79 to 2.60); however, the association did not reach statistical significance ( *p* = 0.292).

**Table 4:** Genotype distributions and allele frequencies of *PON1* polymorphisms among cases and controls

Subject (n)	rs662 ( *575A* *>* *G* )					
	*Genotype distributions (n)*			*Allele frequencies*		OR (95% CI)	*p* -Value
	*AA*	*AG*	*GG*	*A*	*G*	A versus any G	
Controls (50)	26	17	7	0.69	0.31	
Hypothyroidism (50)	34	14	2	0.82	0.18	**2.05 (1.05–3.97)**	**0.047**
Colorectal cancer (50)	26	14	10	0.66	0.34	0.87 (0.48–1.57)	0.762
Pancreatitis (50)	23	24	3	0.70	0.30	1.05 (0.57–1.91)	1.000
	**rs854560 (** ***163T*** ** ** ***>*** ** ** ***A*** **)**					
	***Genotype distributions (n)***			***Allele frequencies***		**OR (95% CI)**	***p*** **-Value**
	***AA***	***TA***	***TT***	***A***	***T***	**A versus any T**	
Controls (50)	20	23	7	0.63	0.37	
Hypothyroidism (50)	20	10	0.60	0.40	0.88 (0.49–1.55)	0.771
Colorectal cancer (50)	17	23	10	0.57	0.43	0.78 (0.44–1.37)	0.471
Pancreatitis (50)	26	19	5	0.71	0.29	1.44 (0.79–2.60)	0.292

Abbreviations: 95% CI, 95% confidence intervals; n, number of subjects; OR, odds ratio; rs, reference SNP number.

Note: *p* < 0.05, significance level.

## Discussion

Genetic polymorphism studies contribute to the generation and identification of gene maps related to various diseases such as cancer, development of new drugs by targeting proteins encoded by specific genes, elucidation of molecular basis of different responses of individuals to drugs, and determination of the factors to be considered in drug selection in treatment. The importance of genetic differences in recent years has attracted the attention of researchers. 30 31 PON1 activity shows different distribution in different ethnic groups. The guanine exchange of cytosine at position 55 in the 3rd exon (rs854560) and the adenine thymine change at position 192 in the 4th exon (rs662) are the most important gene variants in PON1 activity. The *C/G* nucleotide change at position 55 leads to the change of leucine to methionine (Leu55Met, L55M, rs854560) in amino acid sequence, while the changes from glutamine to arginine (Gln192Arg, Q192R, rs662) appear as a result of *A/T* nucleotide change at position 192. 32 33 34

There are some studies related to *PON1* polymorphism in the Turkish population. In a study conducted on a group of individuals with cardiovascular disease, the *G* allele frequency of *PON1* rs662 was higher in the patient group, but no statistically significant difference was found than the healthy individuals. 35 In another study, the relation between cardiovascular diseases and *PON1* rs662 and rs854560 polymorphisms was investigated. The *T* allele frequency of *PON1* rs854560 was higher in the patient group. It is stated that rs854560 polymorphism can be considered as a risk factor for the development of cardiovascular disease among the Turkish population. 27 A study was conducted to determine *PON1* rs662 and rs854560 polymorphisms distribution in diabetic patients with complications and the changes in enzyme activity. Individuals with *TT* genotypes of rs854560 and with *AA* genotypes of rs662 were seen very frequently in type 2 diabetes group; also, enzyme activity was found to be lower in the patients' group. 36 In another study conducted in diabetic patients, a significant relationship between the two polymorphisms and the disease was determined. PON1 enzyme, which is heterozygote for rs662, was found to be three times more effective in the occurrence of complications; also, it was found to be more effective in relation to the risk of diabetic complications. 37

It is well-known that higher levels of oxidative stress are one of the main causes for cancer development. In normal conditions, PON1 activity protects the organisms against oxidative stress via ROS detoxification. PON1 activity is highly variable during pathogenesis and its complex regulation involves both genetic and environmental factors. It can be expected that variations on *PON1* gene may cause alterations on the PON1 activity; and, decreased PON1 activity may cause cancers. Stevens et al 38 observed no association between prostate cancer and *PON1* rs662 and rs854560 polymorphisms; however, when they combined these SNPs, they suggested that men with *PON1* genotypes face an increased risk of contracting aggressive prostate cancer. On the other hand, Antognelli et al 39 found that rs662 AG and rs854560 *TA/TT* genotypes had a significantly higher risk of prostate cancer. Most research works have been focused on the effects of *PON1* polymorphisms in breast cancer; however, they have yielded conflicting results. According a meta-analysis conducted by Saadat, 40 *G* allele of rs662 was associated with decreased risk of breast cancer, whereas both *TA* and *TT* genotypes of rs854560 were associated with increased risk of breast cancer. It is reasonable to expect that *PON1* polymorphisms, due to their lipid peroxidation scavenging activity, may influence colorectal cancer development as well. Similar to Van der Logt et al, 24 our results indicated that in *PON1* rs662 and rs854560 polymorphisms have no effect on colorectal cancer susceptibility.

Considering the crucial role of *PON1* in the regulation of the oxidative stress and inflammation, it might be involved in the pathophysiology of acute pancreatitis. Unal et al 41 showed significantly decreased PON1 activity and positive correlation to HDL level, with significantly increased malondialdehyde levels in acute pancreatitis among Wistar albino rats. Since genetic variations may result in altered activity of PON1, Verlaan et al 42 investigated *PON1* variants as a modifying factor of chronic pancreatitis. It was observed that allele frequencies of *PON1* rs854560 did not differ between chronic pancreatitis patients and controls, whereas it was found in subgroup analysis that *PON1* rs662 *A* allele was significantly higher in idiopathic chronic pancreatitis patients compared with controls. Our results are the first in the literature which showed that individuals with *T* allele of rs854560 had an approximately 1.5-fold risk of developing acute pancreatitis; however, the association did not reach statistical significance.

According to some researchers, both hypo- and hyperthyroidism are associated with low-density lipoprotein (LDL) oxidation. 43 44 Yavuz et al 45 reported iatrogenic thyroid hormone excess might affect thyroid hormone excess on PON1 activity. Also, it was reported that dyslipidemia in thyroid diseases and lipid oxidation, due to oxidative stress, played an important role in the pathogenesis of thyroid diseases. Several studies 20 23 46 revealed that serum PON1 activity was significantly decreased in hypothyroidism. Although the mechanism is unclear, several possible mechanisms, which involve increased ROS production and oxidative stress, are suggested. 47 Since the Q to R ( *A* > *G* ) substitution of *PON1* rs662 forms more active PON1 with higher capacity of detoxification, 39 individuals carrying *A* allele should be less vulnerable to oxidative stress products and lipid peroxidation. Our results showed that individuals carrying *G* allele of rs662 had an approximately two-fold risk of developing hypothyroidism (OR = 2.05; 95% CI = 1.05 to 3.97; *p* = 0.047). In accordance to recent knowledge, it can be suggested that *PON1* rs662 polymorphisms had a substantial effect on hypothyroidism pathogenesis.

## Conclusion

The present study is a comprehensive cross-sectional study, and the first study regarding the effects of *PON1,* an oxidative stress-related gene, and polymorphisms on disease susceptibility in a Turkish population diagnosed with colorectal cancer, hypothyroidism or pancreatitis. There are limited studies about the effects of *PON1* polymorphisms on common diseases; thus, it is believed that preliminary results of this study will elucidate the relation. In the present study, a substantial association between *PON1 G* allele of rs662 and hypothyroidism and a weak association between *PON1 T* allele of rs854560 and pancreatitis were identified. No effect of both PON1 variants was observed on colorectal cancer development. Considering the lack of data, further studies with larger number of hypothyroidism patients and detailed biochemical parameters are needed.
